# 

**Published:** 1891-05-02

**Authors:** 


					INVALIDS
for INFANTS
IllJi
)rwich hosputal n
jfjlHOMAS'S HOSPi
itAscof Western Infirmary
WITH EXTRA NURSING SUPPI.EMENT,
Saturday, May 2nd, 1891.

				

